# Gas fiber lasers may represent a breakthrough in creating powerful radiation sources in the mid-IR

**DOI:** 10.1038/s41377-022-00728-5

**Published:** 2022-02-11

**Authors:** Andrey Pryamikov

**Affiliations:** Prokhorov General Physics Institute of the Russian Academy of Sciences, 38 Vavilov street, Moscow, Russia

**Keywords:** Fibre lasers, Photonic crystals

## Abstract

Continuous wave fiber laser created on the basis of silica glass negative curvature hollow core fiber filled with HBr make it possible to obtain efficient narrow linewidth mid-IR emission with a maximum laser power of about 500 mW at wavelength of 4200 nm. It is for the first time that emission from a continuous wave fiber laser have been achieved at a wavelength of 4496 nm with the largest tuning range of 686 nm.

It is well known that creation and development of discrete and tunable sources of the mid - IR laser radiation has a very long history. The basis of mid-IR photonics was laid when creating quantum cascade lasers^1^, vibronic-state lasers^2^ and optical parametric oscillators^3^. All these types of solid- state mid-IR lasers have their advantages and disadvantages. For example, quantum cascade lasers which were built out of quantum semiconductor structures can cover the spectral range from the mid-infrared to the sub-millimiter spectral region in the continuous wave (CW) mode. But, the heat issue generates serious challenges for creating of high-power mid-infrared light sources. Although the other above-mentioned solid-state laser sources in the mid-IR spectral range have an efficiency sufficient for commercial applications, significant disadvantages^4^ such as a narrow linewidth and linear polarized excitation for parametric generation are still widely exists.

Over the last decade, the development of high-power fiber lasers operating in the mid-infrared spectral range has grown massively, partly thanks to a rapidly maturing technology of soft-glass fiber fabrication^5^. Optical fibers made of soft glasses have significantly lower phonon absorption in the mid-IR spectral range compared to optical fibers made of silicate glass or rare-earth-doped silica glass fibers and, accordingly, significantly lower material losses^6^. This factor makes it possible to actively use them to create power fiber lasers in the mid-IR^4^. Fiber lasers based on fluoride glasses have proved particularly successful^7^. For example, erbium - doped fluorozirconate glass fibers allowed to generate 30 Watt mid-infrared laser output power at a wavelength near 3 μm^8^ and watt-level fiber laser output power at wavelengths up to 3.55 μm^9^ (Fig. 1). The longest wavelength generation in the mid-IR from the fluoride fiber laser was obtained at a wavelength of 3.92 μm using a heavily holmium-doped fluoroindate fiber at CW output power of 200 mW^7^ (Fig. 1). Despite such an impressive result, since then no new record values of CW output power at wavelengths at and beyond of 4 μm have been obtained in fluoride fiber lasers which is primarily due to high quantum defect, thermal management and fiber failure^10^.

**Fig. 1:**
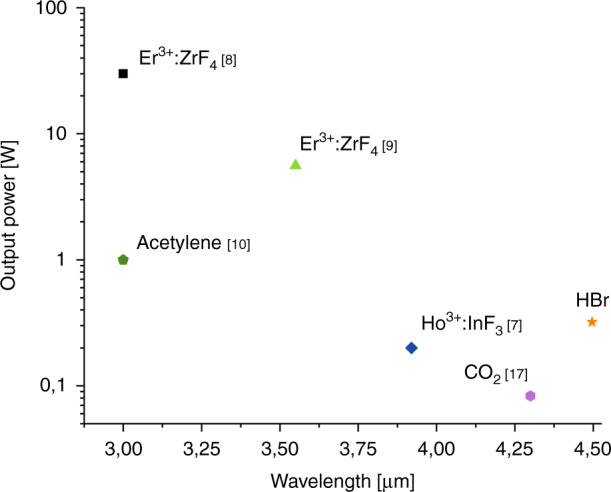
Continuous wave mid-IR fiber lasers. Summary of the state-of-art continuous wave mid-IR fiber lasers in terms of output and lasing wavelengths.

The situation in the field of fiber lasers operating in the mid-IR spectral range began to change dramatically with the development of hollow core fiber technology. This was especially evident after the creation of the so called “negative curvature” or “anti-resonant” hollow core fibers^11–13^ (Fig. 2). They had promising optical properties which allowed to transmit light in the mid-IR spectral range even in fibers made of silica glass^14^. The transmission spectrum of these hollow core fibers with a cladding consisting of capillaries has a band structure. The interaction of low order air core modes is extremely small with the cladding at a sufficiently large value of the air core diameter near the centers of the transmission bands^15^. The new type of gas-filled hollow core fiber lasers created on their basis and made of silica glass were devoid of the disadvantages that were listed above for fluoride fiber lasers. This led to the creation of sufficiently efficient fiber lasers the principle of operation of which is based on two physical mechanisms, namely, population inversion realized by intrinsic absorption of gas molecules^10,16,17^ (Fig. 1) and the stimulated Raman scattering^18,19^. It should be taken into account that Raman lasers based on the hollow core micro-structured fibers have a threshold which is about 5–6 orders of magnitude higher than that based on the population inversion, which leads to all-reported Raman lasers being pulsed.

**Fig. 2:**
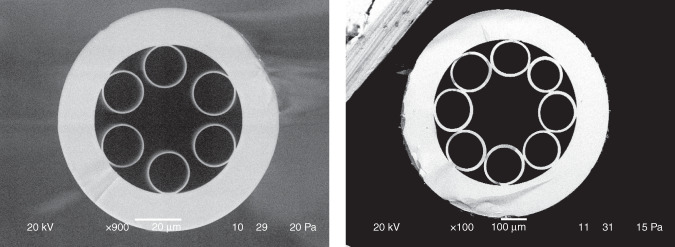
Cross - sections of the negative curvature hollow core fibers made of silica glass (left) and chalcogenide glass (right). The fibers were fabricated at Dianov Fiber Optics Research Center.

In a paper recently published on Light: Science and Applications, the authors set a task of creating a CW fiber laser based on silica glass hollow core negative curvature fiber operating with the highest possible output power at wavelengths greater than 4 μm in the broadest tuning range^20^. For these purposes, they used a population inversion realized by intrinsic absorption of HBr molecules. In their previous work^17^ they used CO_2_ filled hollow core fiber but the possible output laser wavelength range was small enough (Fig. 1) due to the transition properties of CO_2_ molecules. In their new work, the authors used a narrow linewidth 2 μm thulium-doped fiber amplifier seeded by a group of fine-tunable diode lasers to pump a 5-meter-long silica glass hollow core fiber with a cross-section similar to the cross-section of the hollow core fiber shown in Fig. 2 (left). The hollow core fiber was filled with low –pressure HBr gas. The maximum laser output of 500 mW was achieved at wavelength of 4.2 μm with tuning range of 686 nm when the HBr pressure was 5 mbar. Also, the laser output of about 300 mW was achieved at the longest wavelength of 4.496 μm among CW fiber lasers (Fig. 1). Further research in this field, according to the authors of the paper, is connected with two main directions, namely, an employment of all-fiber structure coupling with low loss between hollow core fibers and solid- core fibers and also achieving high power output.

The situation can also be changed with creation of the hollow core fibers made of non-silica glasses made of tellurite glasses or chalcogenide glasses^21–23^. Their use will significantly reduce material losses of the fiber cladding in the mid -IR spectral range to obtain CW laser generation at wavelengths greater than 4.5 μm. To date, tellurite hollow core fibers have been developed by an extrusion and draw approach. In^22^ the fiber losses of 4.8 and 6.4 dB/m were measured at 5.6 and 5.8 µm, respectively. According to the authors^22^, this gives hope that in the near future it will be possible to fabricate tellurite hollow core optical fibers with sub 1–2 dB/m loss anywhere in the technologically important spectral region between 4.5 and 6.5 µm. In^23^ the authors reported the fabrication of a hollow core fiber drawn from chalcogenide glass 3D printed preform. This fiber showed several transmission bands in the 2–12 µm spectral range.

In conclusion, CW gas fiber lasers based on the new type of hollow core fibers are becoming an emerging competitor to their rare–earth–doped counterparts in the mid–IR spectral range. It is quite possible that in the near future they will become a good alternative compared to other types of fiber lasers, for creating CW coherent light sources in the mid–IR spectral range.
